# Increased Oral Dryness and Negative Oral Health-Related Quality of Life in Older People with Overweight or Obesity

**DOI:** 10.3390/dj10120231

**Published:** 2022-12-06

**Authors:** Nattapat Khongsirisombat, Sirichai Kiattavorncharoen, Supanee Thanakun

**Affiliations:** 1 Geriatric Dentistry Clinic, Dental Hospital, Faculty of Dentistry, Mahidol University, Bangkok 10400, Thailand; 2 Faculty of Dentistry, Western University, Pathumthani 12000, Thailand; 3 Department of Oral and Maxillofacial Surgery, Faculty of Dentistry, Mahidol University, Bangkok 10400, Thailand; 4 Division of Oral Diagnostic Science, College of Dental Medicine, Rangsit University, Pathumthani 12000, Thailand

**Keywords:** obesity, oral health-related quality of life, oral health status, xerostomia

## Abstract

This cross-sectional study was to evaluate the association between the oral health-related quality of life (OHRQoL) of older Thai people with obesity and oral health indicators. General and oral conditions were assessed. Oral dryness was determined using the Xerostomia Inventory-11 (XI-11) and clinical oral dryness score (CODS). OHRQoL was evaluated by the oral health impact profile (OHIP-14). Participants were aged 60–86 years; 73 (59.3%) were overweight or obese, and 50 (40.7%) were normal weight. Older patients with obesity had almost four times the rate of hypertension (OR = 3.59; 95%CI:1.34–9.60; *p* = 0.002), more missing teeth (*p* = 0.025), and higher CODS (*p* = 0.014) than those without obesity. The total XI-11 scores were positively associated with the total CODS, after adjusting for BMI (*r* = 0.267, *p* = 0.003). Those with obesity had almost three times the tendency for a negative OHRQoL compared with the non-obese (OR = 2.73; 95%CI:1.12–6.71; *p* = 0.04). After adjusting for all related factors, the chances of predicting an OHIP-14 score of four based on obesity and total XI-11 score were 4.42 (95%CI:1.57–12.47; *p* = 0.005) and 1.11 (95%CI:1.02–1.20; *p* = 0.013), respectively. Obesity had an increasingly undesirable negative impact on the OHRQoL of older Thai people and was influenced by BMI and oral dryness.

## 1. Introduction

A rapid demographic transition characterized by increasing life expectancy and an aging society in Thailand has been reported [1]. Population aging is driven mainly by improved living conditions and medical advances. The impacts of oral conditions on individuals’ quality of life (QoL) are commonly conceptualized in dental research as oral health-related quality of life (OHRQoL) [2]. This multidimensional construct incorporates individuals’ subjective notions related to functional and emotional well-being, as well as expectations of and satisfaction with health care [2]. Several instruments evaluate OHRQoL in the aged population. The Geriatric Oral Health Assessment Index (GOHAI) and Oral Impacts on Daily Performances (OIDP) are OHRQoL measurements that have been widely used in studies on self-perception of oral health among the older population [3]. However, the most common OHRQoL instrument is the Oral Health Impact Profile (OHIP) in all its forms, which includes the OHIP-49 and short forms such as OHIP-14 [3]. A study by Nammontri validated the OHIP-14 Thai version in 2017 [4].

Studies indicate that lower OHRQoL in older adults is associated with increased age, sex, socioeconomic disadvantage, anxiety or depression, negative self-rated general health, poor oral health, lack of dentures, dry mouth, poor chewing and swallowing function, and irregular dental visits [5,6,7,8,9,10,11,12,13,14]. Poor nutritional status has also a deleterious effect on OHRQoL [5].

Our previous study revealed a significant increase in inflammatory-related oral disease in patients with overweight or obesity [15]. Our search of the literature found no studies that have directly explored OHRQoL in older persons with obesity. Most studies reported OHRQoL in adults or older patients with metabolic syndrome (MS), diabetes mellitus (DM), or chronic kidney disease (CKD) [16,17,18]. The OHRQoL of patients with MS, DM, or CKD whose status had an obese component tended to be negatively impacted [16,17,18]. However, Yamashita et al. investigated OHRQoL in young adults with obesity and found no difference in the OHIP scores between obese and non-obese populations [19]. Similarly, Tengku et al. reported a low impact on schoolchildren’s OHRQoL, regardless of their body mass index (BMI) and the severity of oral disease [20].

Dry mouth is highly prevalent in older adults [21,22], subjectively determined as xerostomia and objectively measured as hyposalivation. Worsening dry mouth, resulting in poorer oral conditions and OHRQoL, has also been demonstrated in previous studies [9,11,23]. However, no previous reports have used both measures for dry mouth and investigated their association with OHRQoL in older patients according to BMI. This study, therefore, investigated the factors associated with poor OHRQoL in a sample of Thai community-dwelling older adults with and without obesity. Additionally, we aimed to elucidate the relationship between OHRQoL and dry mouth among older Thai adults with different BMIs, for whom the cut-off is lower than those of other ethnicities.

## 2. Materials and Methods

### 2.1. Patient Selection

One-hundred and twenty-three participants were found eligible to be included out of seven-hundred and eight older Thai patients who attended the Geriatric Dentistry Clinic, Dental Hospital, Faculty of Dentistry, Mahidol University, between October 2019 and April 2022, and were screened for this cross-sectional study. Subjects included were older people aged > 60 years who had medical profiles documenting the following parameters within the last 6 months: high-density lipoprotein–cholesterol (HDL–C), triglycerides (TG), fasting plasma glucose (FPG), blood pressure (BP), and waist circumference (WC). They also had no history of dental treatment within the last 6 months. The exclusion criteria were patients with a history of radiotherapy or chemotherapy in the previous 3 months or the presence of salivary gland diseases that affect oral dryness. The sample size was calculated based on our previous study at a significant level of 95%, and power of 80% [15]. One-hundred and eight participants were required in this cross-sectional study.

All patients provided written informed consent for the use of their data. The study protocol and consent forms complied with the Declaration of Helsinki and were approved by the Research Ethics Board of the Faculty of Dentistry, Faculty of Pharmacy, Mahidol University. (MU-DT/PY–IRB 2019/048.3107).

### 2.2. Assessment of General Health

Participants were classified into three study groups according to BMI. BMI was calculated based on the patient’s body weight in kilograms (kg) divided by height in meters squared (m^2^). For participants unable to stand upright, height measurement was requested from the participant, or data were taken from the national identification card. A BMI of 18.5–22.9 kg/m^2^ was considered a normal weight for this older Thai population. A BMI of 23.0–24.9 kg/m^2^ indicated overweight, whereas a BMI of ≥25 kg/m^2^ indicated obesity [24]. The cut-off for abnormal laboratory results followed these criteria: (1) elevated TG: ≥150 mg/dL; (2) reduced HDL–C: <40 mg/dL in men or <50 mg/dL in women; (3) elevated BP: ≥140 mm Hg systolic BP or ≥90 mm Hg diastolic BP; (4) elevated FPG: ≥100 mg/dL; (5) increased WC: ≥90 cm in men or ≥80 cm in women [25].

### 2.3. Assessment of Patients’ Data

Data from the interviews (by N.K.) included age, sex, medication, education level (higher/lower bachelor’s degree), living status, travel type for dental treatment (independent or dependent), and general and dental health care financial status. Data about personal habits were also collected, including smoking and alcohol consumption (never, former (used to smoke tobacco or drink alcohol but stopped at least 6 months previous to data collection), or current), exercise (e.g., ≥30 min of aerobic exercise ≥3 times per week), and sleeping time (hours).

### 2.4. Assessment of Oral Health

Dental disease data (by N.K.) included dental caries, broken teeth, tooth wear, and pulpal and periapical tissue disease. Missing teeth were also counted. The remaining teeth included sound teeth and retained roots. The third molar was discounted from the study.

The Periodontal Screening and Recording (PSR) index evaluated periodontal status. The method is based on three periodontal disease indicators: gingival bleeding on probing, calculus accumulation, and probing depth. The mouth is divided into six sextants (teeth 18–14, 13–23, 24–28, 34–38, 33–43, and 44–48). Each tooth is examined and coded from 0 to 4 but only the highest score of the sextant is recorded. PSR code definitions are: 0 indicates the absence of any clinical signs, 1 indicates bleeding on probing, 2 indicates supra and/or subgingival calculus and/or defective margins, 3 indicates periodontal pockets 4–5.5 mm in depth, and 4 indicates periodontal pockets 6 mm or more in depth [26]. Sextants with fewer than two teeth are scored with an ‘X’ and are not considered in the overall evaluation [26]. PSR scores of 3 in two or more sextants or a PSR score of 4 in any sextant is diagnosed as periodontitis [26].

Oral mucosal dryness was evaluated with the Xerostomia Inventory (XI-11) questionnaire [27] and the clinical oral dryness score (CODS) [28]. Participants were interviewed face-to-face by one investigator. The answer to each question of the XI-11 (Thai-version) showed the frequency of symptoms in the preceding 4 weeks. The Likert-scale response to the XI-11 questionnaire is in the range of 11–55; a higher total score represents more severe xerostomia than a lower score [27]. Hyposalivation was examined and determined using the CODS. A total of 10 clinical features of CODS are: (1) mirror sticks to buccal mucosa; (2) mirror sticks to tongue; (3) lobulated/fissured tongue; (4) tongue shows loss of papillae; (5) frothy saliva; (6) no saliva pooling in floor of mouth; (7) glassy appearance of the oral mucosa, especially palate; (8) debris on palate; (9) altered/smooth gingival architecture; (10) cervical caries (>2 teeth). Each feature observed contributes a score of 1, giving a total score from 1 to 10. A high total score indicates severe oral dryness [28].

### 2.5. Assessment of Oral Health-Related Quality of Life

The Oral Health Impact Profile (OHIP)-14 developed by Slade was used to determine OHRQoL [29]. The validated Thai version of the OHIP-14 was used in this study [4]. Participants were interviewed face-to-face (by N.K.). The answer to each question showed the perceived frequency in the preceding 4 weeks. The OHIP-14 consists of seven domains, each containing two questions. Responses to the 14 items were graded on a Likert-type scale ranging from 0 to 4: 0 = “never”, 1 = “hardly ever”, 2 = “occasionally”, 3 = “fairly often”, and 4 = “very often” [4,29]. Two aspects of the OHIP-14 were analyzed: prevalence and severity. Prevalence is the percentage of participants responding with a score of 4 for at least one item [30]. Severity is the total score of the Likert scale response to the OHIP-14, with a maximum score of 56 [30]. A higher total score negatively impacted QoL more than a lower score.

### 2.6. Statistical Analysis

Statistical analyses were performed using SPSS software (IBM SPSS Statistics version 26.0; SPSS Inc., Chicago, IL, USA) (by S.T.). Descriptive statistics were used to evaluate data in older people according to BMI status. Fisher’s Exact Test, Pearson’s chi-square test, Spearman’s correlation, the Kruskal–Wallis test, and the Mann–Whitney U test were used where appropriate. Multivariate analysis was applied to determine the factors related to OHRQoL. The responses, dichotomized by the cut-off point of scoring 4 “very often” to determine negative OHRQoL, were used as the dependent variable. Significant factors from clinical relevance or bivariate statistical analysis were put into the model as independent variables. A value of *p* < 0.05 was set for significant results.

## 3. Results

### 3.1. Characteristics of the Study Population

The 123 participants were aged 60–86 years of age. Forty-five participants (36.6%) were classified as obese and twenty-eight (22.7%) were overweight. There were no significant differences in age and sex among the three studied groups. General health profiles (e.g., HDL–C, TG, and systolic BP levels) significantly differed between older people with normal weight and those who were overweight or obese. BMI was significantly correlated with waist circumference (*r* = 0.831, *p* < 0.001). Additionally, there was a significant association between BMI status and hypertension (HT) (*p* = 0.002). Older patients with obesity were almost four times as likely to have HT (odds ratio (OR) = 3.59; 95%CI: 1.34–9.60; *p* = 0.002). There were significant associations between patients with obesity and increased medication use (*p* = 0.001) and medication for HT (*p* ≤ 0.001) with a three-times higher risk (OR = 3.21; 95%CI: 1.36–7.55; *p* = 0.002) and five-times higher risk (OR = 5.23; 95%CI: 2.22–12.29; *p* = 0.001), respectively, (Table 1). The three studied groups recorded no differences in education, finance, living, exercise, or personal habits (Table S1).

### 3.2. Characteristics of Oral Condition

Participants with obesity had more missing teeth than individuals with normal weight or overweight (*p =* 0.025). Total CODS in the obese group was significantly higher than in the other two groups (*p =* 0.014). Nevertheless, the extent of dental disease, periodontal status, denture wearing, and average XI-11 scores were not different among these three groups (Table 2).

#### Oral Mucosal Dryness

Although the average CODS was considerably higher in patients with obesity than in those without obesity, as shown in Table 2, oral mucosal dryness as determined by xerostomia, represented by the total XI-11 score, was not significantly different among the researched groups (*p =* 0.927). However, a positive association of the total XI-11 scores with the total CODS was significant without the influence of BMI (*r* = 0.267, *p* = 0.003) [Figure S1a].

Although the highest percentage of participants with a CODS score of 0 or no signs of dry mouth were from the overweight group, a higher percentage of obese participants exhibited signs of oral dryness ranging from one to six items. A higher CODS score was more prevalent in the obese group than in the non-obese group [Figure S2a]. A similar trend occurred in the mean score of CODS; in most items, participants with obesity had a higher mean oral dryness score than participants without obesity [Figure S2b]. These results demonstrate that participants with obesity had more significant signs of dry mouth as determined by CODS than non-obese participants.

For the prevalence of xerostomia, compared with patients with normal weight, a higher percentage of participants with obesity experienced xerostomia [Figure S3a]. The two highest categorical summations representing more severe xerostomia belonged to participants with obesity [Figure S3a]. The highest scores for seven of the eleven items (63.6%) of the XI-11 were in patients with obesity [Figure S3b]. There was also a significant difference between mean XI-11 scores according to sex (*p* = 0.003). Females (18 (11, 35)) had higher average xerostomia scores than males (15 (11, 42)). A positive correlation of the total XI-11 score with age (*r* = 0.192, *p* = 0.034), systolic BP (*r* = 0.244, *p* = 0.007), and the number of missing teeth (*r* = 0.253, *p* = 0.005) was exhibited without the influence of BMI.

### 3.3. Oral Health-Related Quality of Life

When the total OHIP-14 score was divided into different intervals of severity, it was demonstrated that the percentage of participants with obesity in most categories except the lowest one (score range 0–4) was higher than that in participants with normal weight (Figure S4). Among the 14 items (the seven domains of the OHIP-14), the mean scores of each item in the obese group were higher than those of the non-obese group. The average score was highest for domain 2 (physical pain) in all studied groups [Figure S5a]. Considering each item separately, a trend was observed for the highest mean scores to be recorded in the obese group [Figure S5b]. Thus, the participants with obesity experienced a more deleterious effect on their OHRQoL than participants without obesity.

### 3.4. Association of OHIP-14 with Relevant Factors

OHIP-14 scores of four were associated with BMI status (Table 3). Older participants with obesity were almost three times more likely to have a negative OHRQoL compared with those without obesity (OR = 2.73; 95%CI: 1.12–6.71; *p* = 0.04). Moreover, there was still an association between older participants categorized as overweight or obese according to waist circumference and OHIP-14 scores of four (OR = 2.53; 95%CI: 1.12–5.74; *p* = 0.04). Notably, older people with levels of HDL–C lower than the standard value also had a higher risk of experiencing a negative OHRQoL (OR = 2.83; 95%CI: 1.01–7.38; *p* = 0.04). Consequently, a substantial negative impact on the QoL evaluated by the OHIP-14 tended to be more prevalent in participants with obesity or low HDL–C than in patients with normal weight.

After controlling for BMI, correlations were found between OHIP-14 severity scores and total CODS (*r* = 0.227, *p* = 0.012) [Figure S1b], total XI-11 score (*r* = 0.498, *p <* 0.001) [Figure S1c], number of missing teeth (*r* = 0.231, *p* = 0.011) [Figure S1d], and pulpal disease (*r* = 0.223, *p =* 0.014). Therefore, if older participants had severe oral dryness or a higher number of missing teeth, the impact on their OHRQoL would likely be higher.

Logistic regression analysis finally assessed the variables associated with OHIP-14 scores of four for at least one item (Table 4). After adjusting for all related factors, the OR values for OHIP-14 scores of four as predicted by obese status and total XI-11 scores were 4.42 (95%CI: 1.57–12.47; *p* = 0.005) and 1.11 (95%CI: 1.02–1.20; *p* = 0.013), respectively, (Table 4). The significant factors have led to the postulation of the following prognostic equation. For every point of BMI or 1 cm increase in waist circumference, the chance of unfavorable OHRQoL will also increase by a factor of 1.23 (OR 1.23: 95%CI: 1.08–1.40; *p* = 0.001) or 1.06 (OR 1.06: 95%CI: 1.01–1.11; *p* = 0.021), respectively, without the influence of xerostomia. Additionally, for every additional item in the XI-11 score, the risk of a negative impact on OHIP-14 scores of four will increase by a factor of 1.12 (OR 1.12: 95%CI: 1.04–1.20; *p* = 0.003), without the influence of BMI.

## 4. Discussion

To the best of our knowledge, this is the first study to report that obesity and oral dryness have a deleterious effect on QoL in older people after adjusting for age and sex. Although some studies on obesity were found, they mainly investigated young adults or schoolchildren and found that the impact on OHRQoL was insignificant regardless of their BMI and the severity of oral disease [19,20]. Dry mouth has not often been studied in relation to OHRQoL and is usually investigated in the general older population, not taking BMI into account [3]. Older adults in Brazil with a history of chronic disease and negative perceptions of their general health exhibited a significantly higher prevalence of poor OHRQoL [6,13]. In the present study, we recruited 123 Thai people aged over 60 years. The ratio of older patients with overweight or obesity was comparable with the prevalence of older people with obesity in studies of the general Thai population [31,32]. The median age of older participants was matched, with no difference between the sexes and with no negative effect on OHRQoL, which is in contrast with the studies of Lindmark et al. [12] and Oliveira et al. [13]. BMI, WC, TG, and systolic BP were higher in the obese group than in the non-obese group, confirming the recruitment of participants for the study who experienced adverse impacts on OHRQoL. Levels of FPG were not significantly different among all the studied groups, excluding the influence of DM on lower OHRQoL [16].

There was a strong association between participants with obesity and HT and antihypertensive drugs, similar to our previous studies on adult and older Thai people [33,34] and another study [19]. In fact, obesity initiates the development of HT and this pathophysiology has been reported [35]. Several medications, including antihypertensive drugs, have an influential and dose-dependent association with xerostomia [22]. Medication for HT-induced hyposalivation might decrease self-cleansing and enhance dental caries susceptibility, leading to pulpal disease [36]. Generally, apart from experiencing xerostomia, participants with dental disease, especially pulpal disease, may have a higher chance of experiencing pain that impacts QoL. It is notable that all groups in the present study had the highest frequency of responses for item 3 (pain) and item 4 (uncomfortable to eat) of the OHIP-14 (domain 3), which indicates why the higher rate of dental disease in patients with obesity might be a factor related to OHRQoL.

The personal data of participants in all groups were similar. The obese participants’ median sleep duration was 6 h, which was less than the recommended sleep duration for older people (>7 h) [36,37,38]. Sato et al. demonstrated that people with low QoL determined by the OHIP-14 exhibited poor sleep quality [36]. Therefore, participants with obesity may face adverse QoL due to insufficient sleep. However, this study did not record the use of sleep medications such as hypnotics, anxiolytics, or antidepressant drugs, which are frequently used by older people. Future sleep medication data related to sleep quality and duration might better indicate an association between sleeping and QoL. Additionally, a negative influence of smoking on OHRQoL has been documented [6,14], which is inconsistent with our findings. This may have been a reflection of the small number of participants who smoked in the present study.

While the latest systematic review presents an association between obesity and periodontitis, most of the included studies adjusted the results according to age [39]. The results of our study demonstrated that periodontal status was not different among all the older subjects, which is consistent with previous research that found no difference in periodontitis between older obese and non-obese groups [40]. The teeth with periodontitis may have been extracted, and the remaining teeth exhibited no severe periodontal or other dental diseases. Additionally, the number, characteristics of participants, and the criteria of the periodontal examination might be responsible for the absence of an association between periodontitis and obesity in this study. A recent systematic review established an association between periodontal disease and poor OHRQoL among older persons [14]. Cortelli et al. also found that deteriorated periodontal parameters were associated with worse QoL in obese patients [41]. If people with obesity exhibit periodontitis, non-surgical periodontal therapy will lower circulated inflammatory mediators that could lead to future cardiovascular disease [42]. Therefore, periodontal therapy may be essential in improving the OHRQoL of obese individuals [41].

There was a significant difference in the number of missing teeth between participants with and without obesity in this study. After controlling for BMI, a positive correlation was found between the OHIP-14 and several missing teeth. A higher number of missing teeth was associated with lower OHRQoL in patients with obesity. Our result was similar to a previous Thai survey, which reported that Thai elders with less than 20 natural teeth or less than four posterior occluding pairs, had lower OHRQoL than individuals with a minimum of twenty natural teeth or at least four posterior teeth occluding pairs [43]. Recent research found that the OHRQoL of older Chinese or Korean elders was mainly affected by the loss of teeth [7,8]. Nevertheless, the correlation in the current study disappeared after other influential variables were adjusted in the multivariate analysis. Another study similarly found that the number of missing or remaining teeth had no impact on OHRQoL [44]. However, the number of occluding pairs and the location of the remaining teeth have a major impact on OHRQoL. It is possible that the average number of remaining teeth in participants with obesity in the current study was still higher than 20. One study reported an increase in OHRQoL 3 months after the insertion of new removable dentures [9]. Further study on this controversial topic and the necessity for wearing a prosthesis in a larger sample of older people would clarify the correlation with OHRQoL.

Dry mouth was assessed with the XI-11 questionnaire to determine xerostomia and CODS to evaluate clinical signs of hyposalivation without direct salivary flow measurement. The number of obese and non-obese older participants with xerostomia as determined by the XI-11 questionnaire was similar in this study, which is in contrast with the study by Torres et al. [45]. Nevertheless, in the final statistical analysis of the present study, xerostomia was a significantly important factor influencing negative OHRQoL regardless of age, sex, or BMI. These results were comparable with those of a study by Choi et al., which found that the more patients perceived their saliva amount to be insufficient, the lower their reported OHRQoL [46]. Other studies have also reported that deteriorating dry mouth results in lower OHRQoL in older adults [9,11,23,36]. Notably, the present study revealed a correlation between xerostomia and CODS after controlling for BMI. Most participants with normal weight had a total CODS score of 0. In contrast, the total CODS scores of the obese group varied from one to six, while no participants in the non-obese group had a total score of six. Jager et al. confirmed that CODS was associated with the XI, decreased unstimulated and stimulated salivary flow, and the Bother Index (BI) [47]. The current results show a comparable pattern: a difference in CODS among the studied groups was established despite no differences in xerostomia. Salivary flow reduction is not necessarily associated with the subjective feeling of dry mouth and other xerostomia problems [21]. Ohara et al. showed that although no difference was observed between hyposalivation and the severity of physical frailty, xerostomia is associated with physical frailty, which is defined as having three or more components of weakness, slowness, weight loss, low physical activity level, or exhaustion among older adults adjusted for demographic and health characteristics [48]. Older patients perceived that their xerostomia and OHRQoL improved after receiving new removable partial or complete dentures [9].

The number of signs of dry mouth (i.e., CODS) was more significant in the obese group than in the non-obese group. Older people often have complex systemic diseases and use multiple medications. Drug-induced hyposalivation might occur in this group, often with the use of antihypertensive drugs [49]. Hyposalivation in older people was verified in a systematic review, which concluded that the overall prevalence of hyposalivation in older people is 33.4% [21]. Furthermore, the salivary flow rate was lower in individuals with obesity than in normal-weight persons [19]. In our study, most of the older participants with obesity had HT and used antihypertensive drugs that can cause hyposalivation. The self-adaptation of participants with obesity might explain the differences in results between the XI-11 and CODS in the obese and non-obese groups. Due to self-adaptation, participants might also have hyposalivation with no sensation of dry mouth. Individuals might slowly adjust themselves to their dry mouth sensation because of its steady prolonged duration. At some point, they may experience a decreased saliva flow rate, leading to a dry mouth sensation. Jager et al. suggested that XI, CODS, and BI can differentiate between hyposalivation and normal salivation because participants in their study became aware of dry mouth symptoms when the salivary flow dropped below 0.1 mL/min [47]. Accordingly, they recommend using CODS or a combination of CODS with XI or BI as a routine clinical assessment to detect hyposalivation [47]. Participants with obesity should beware of oral problems due to hyposalivation despite the absence of xerostomia symptoms. Further studies in a larger population should clarify these conflicting results.

The highest mean score in the OHIP-14 in all studied groups was in domain 2 for the item of physical pain. Domain 2 is determined by pain and discomfort when eating. Our results are consistent with previous studies in Norway that revealed that older people’s most frequently experienced problems were with pain in the mouth and discomfort when eating [10]. It was not surprising that oral pain leads to a negative OHRQoL. Older adults experience difficulty chewing and swallowing due to dry mouth, missing teeth, and dental and periodontal problems that result in discomfort while eating and drinking [11,46]. The next highest mean scores in the OHIP-14 were for the items of unsatisfactory diet and less satisfaction with life in the current study. Effective pain management or prevention of oral problems in older people may improve OHRQoL. Additionally, regular dental visits and treatment of oral disease can prevent weight gain and poor self-perception of oral health [45].

This study had some limitations. A cross-sectional survey does not yield cause–effect interpretations. The small number of participants caused by the COVID-19 pandemic and restrictions on health services by the Thai government necessitated several interruptions to this research. Furthermore, numerous participants were excluded from our study because they had no laboratory results or had a history of recent dental treatment. Specific factors that might be associated with obesity and xerostomia, such as occupation and income level, were not considered. Our findings may not be generalizable; therefore, these results should be cautiously interpreted. Despite these limitations, this study highlights the possible impact of obesity and xerostomia on OHRQoL after adjusting for potential confounders. This was the first study to demonstrate a relationship between unfavorable OHRQoL and older patients with obesity in Thailand. Future studies should be performed with a larger sample size to collect additional data about occlusion and nutrition. A longitudinal study may demonstrate the underlying reasons for the deterioration in OHRQoL in older people with overweight or obesity.

## 5. Conclusions

General and oral health are significant components of OHRQoL in older adults. Obesity and dry mouth negatively impact the QoL in Thai older people without the influence of various confounders. Oral health practitioners should be aware that evaluation and management of dry mouth, both xerostomia and hyposalivation, benefit the QoL of older dental patients, as does routine dental treatment regardless of their BMI status.

## Figures and Tables

**Table 1:** Characteristics of participants according to body mass index (BMI) (n (%) or median (first, third quartile)).

	Participants (N = 123)			*p*-Value
	Normal Weight (n = 50)	Overweight (n = 28)	Obesity (n = 45)	
Age	65 (63, 68)	66 (62, 69)	65 (61, 69)	0.767
Sex				
Male	25 (50.0)	14 (50.0)	24 (53.3)	0.939
Female	25 (50.0)	14 (50.0)	21 (46.7)	
BMI (kg/m^2^)	21.1 (20.2, 22.0)	24.2 (23.4, 24.8)	27.6 (26.2, 29.6)	<0.001 *
WC (cm)				
Male	80 (78, 82)	89 (87, 91)	97 (90, 102)	<0.001 *
Female	75 (68, 78)	83 (79, 87)	87 (84, 96)	<0.001 *
FPG (mg/dL)	100 (95, 112)	100 (92, 117)	104 (96, 115)	0.335
HDL (mg/dL)				
Male	55 (47, 66)	57 (52, 70)	51 (45, 57)	0.040
Female	75 (64, 81)	62 (46, 74)	52 (47, 61)	<0.001 *
TG (mg/dL)	79 (63, 121)	97(7, 134)	116 (86, 178)	<0.001 *
Systolic BP (mmHg)	121 (111, 131)	132 (119, 138)	128 (121, 141)	0.017 *
Diastolic BP (mmHg)	76 (64, 84)	78 (70, 88)	79 (74, 87)	0.228
Hypertension criteria				
Optimal HT <120 and/or <80 mmHg	24 (48.0)	6 (21.4)	7 (15.6)	0.002 *
Normal 120–129 and/or 80–84 mmHg	7 (14.0)	5 (17.9)	16 (35.5)	
High normal 130–139 and/or 85–89 mmHg	13 (26.0)	8 (28.6)	7 (15.6)	
Possible hypertension >140/90 mmHg	6 (12.0)	9 (32.1)	15 (33.3)	
Number of medication use				
No	28 (56.0)	9 (32.1)	13 (28.9)	0.001 *
1 group	13 (26.0)	12 (42.9)	8 (17.8)	
≥2 groups	9 (18.0)	7 (25.0)	24 (53.3)	
Medication for diabetes mellitus				
No	42 (84.0)	26 (92.9)	37 (82.2)	0.430
Yes	8 (16.0)	2 (7.1)	8 (17.8)	
Medication for dyslipidemia				
No	31 (62.0)	16 (57.1)	18 (40.0)	0.088
Yes	19 (38.0)	12 (42.9)	27 (60.0)	
Medication for hypertension				
No	41 (82.0)	14 (50.0)	20 (44.4)	<0.001 *
Yes	9 (18.0)	14 (50.0)	25 (55.6)	

* Significance level at *p* < 0.05 (Kruskal–Wallis test, Mann–Whitney U test, or λ^2^).

**Table 2:** Characteristics of the oral condition of participants according to body mass index (BMI) (median (first, third quartile) or n (%)).

	Participants (N = 123)			*p*-Value
	Normal Weight (n = 50)	Overweight (n = 28)	Obesity (n = 45)	
Number of tooth diseases	9 (5, 13)	10 (7, 15)	11 (5, 16)	0.366
Number of missing teeth	3 (1, 5)	3 (0, 5)	5 (2, 9)	0.025 *
Periodontal status				
Gingivitis	39 (78.0)	17 (60.7)	29 (64.4)	0.198
Periodontitis	11 (22.0)	11 (39.3)	16 (35.6)	
Denture wear				
Upper	11 (22.0)	7 (25.0)	11 (24.4)	0.421
Lower	7 (14.0)	5 (17.9)	8 (17.8)	0.171
Total XI-11 score	17 (12, 21)	17 (14, 20)	17 (15, 22)	0.927
Total CODS	0 (0, 2)	0 (0, 2)	2 (0, 3)	0.014 *

* Significance level at *p* < 0.05 (Kruskal–Wallis test, Mann–Whitney U test).

**Table 3:** Prevalence of participants (n (%)) who scored four for at least one item on the oral health impact profile (OHIP-14). * Significance level at *p* < 0.05 (λ^2^).

OHIP-14	Participants (N = 123)			*p*-Value
	Normal Weight (n = 50)	Overweight (n = 28)	Obesity (n = 45)	
Score 0–3	42 (84.0)	23 (82.1)	25 (55.6)	0.004 *
Score 4	8 (16.0)	5 (17.9)	20 (44.4)	

**Table 4:** Logistic regression analysis for oral health-related quality of life (OHRQoL) and associated factors (odds ratio (95% confidence interval)) (n = 123).

	OHIP-14: Scoring 4							
	Model 1		Model 2		Model 3		Model 4	
	OR (95%CI)	*p*	OR (95%CI)	*p*	OR (95%CI)	*p*	OR (95%CI)	*p*
Age (years)	1.03 (0.95, 1.12)	0.492	1.02 (0.94, 1.11)	0.631	0.98 (0.88, 1.09)	0.672	0.96 (0.86, 1.08)	0.541
Sex (female)	1.38 (0.62, 3.07)	0.434	1.49 (0.64, 3.48)	0.352	1.64 (0.69, 3.94)	0.266	1.29 (0.51, 3.22)	0.593
BMI								
Overweight			1.13 (0.33, 3.88)	0.846	1.24 (0.35, 4.34)	0.737	1.41 (0.39, 5.08)	0.603
Obesity			4.25 (1.62, 11.15)	0.003	4.06 (1.51, 10.90)	0.005	4.42 (1.57, 12.47)	0.005 *
Number of missing teeth					1.05 (0.95, 1.15)	0.326	1.03 (0.93, 1.15)	0.570
Number of teeth with pulpal diseases					1.26 (0.89, 1.79)	0.194	1.24 (0.79, 1.95)	0.353
Total XI score							1.11 (1.02, 1.20)	0.013 *

* Significance level at *p* < 0.05 (Logistic regression analysis).

## Data Availability

The data are not publicly available due to ethical restrictions and are available on request from the corresponding author.

## Supplementary Materials

The following supporting information can be downloaded at: https://www.mdpi.com/article/10.3390/dj10120231/s1: Table S1: Family, social, and personal history of participants according to BMI; Figure S1: Correlation of Xerostomia Inventory-11 (XI-11) scores with the clinical oral dryness score (CODS); the severity of the oral health impact profile (OHIP-14) with CODS, XI-11 score, and number of missing teeth; Figure S2: Percentage of participants according to the total clinical oral dryness score (CODS) and mean CODS according to each item; Figure S3: Percentage of participants according to total Xerostomia Inventory-11 (XI-11) score and mean XI-11 score according to each item; Figure S4: Percentage of participants according to the oral health impact profile (OHIP-14) total score; Figure S5: The average severity score of the oral health impact profile (OHIP-14) according to 7 domains and 14 items.
