# Supplementary material for: Increased Oral Dryness and Negative Oral Health-Related Quality of Life in Older People with Overweight or Obesity
Source: Dent J (Basel). 2022 Dec 6;10(12):231. doi: 10.3390/dj10120231 (PMC9776969; doi:10.3390/dj10120231)
Supplement: Supplementary file 1 [file dentistry-10-00231-s001.zip › Figure S1.pdf]

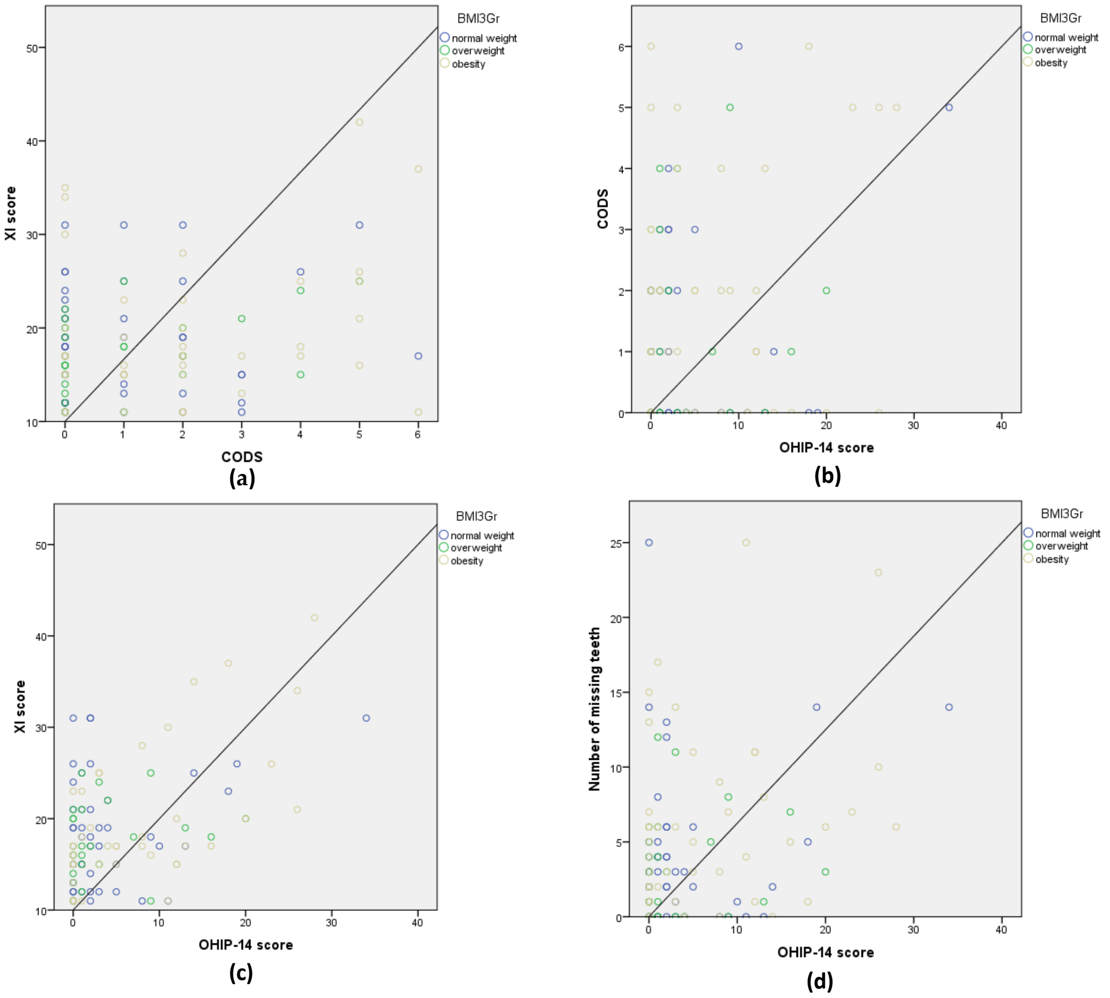

Figure S1. (a) Correlation of Xerostomia Inventory-11 (XI-11) scores with the clinical oral dryness score (CODS); **(b)** the severity of the oral health impact profile (OHIP-14) with CODS; **(c)** the severity of the OHIP-14 with XI-11 score**; (d)** the severity of the OHIP-14 with number of missing teeth; adjusted for body mass index (BMI).
