# Supplementary material for: Increased Oral Dryness and Negative Oral Health-Related Quality of Life in Older People with Overweight or Obesity
Source: Dent J (Basel). 2022 Dec 6;10(12):231. doi: 10.3390/dj10120231 (PMC9776969; doi:10.3390/dj10120231)
Supplement: Supplementary file 1 [file dentistry-10-00231-s001.zip › Figure S2.pdf]

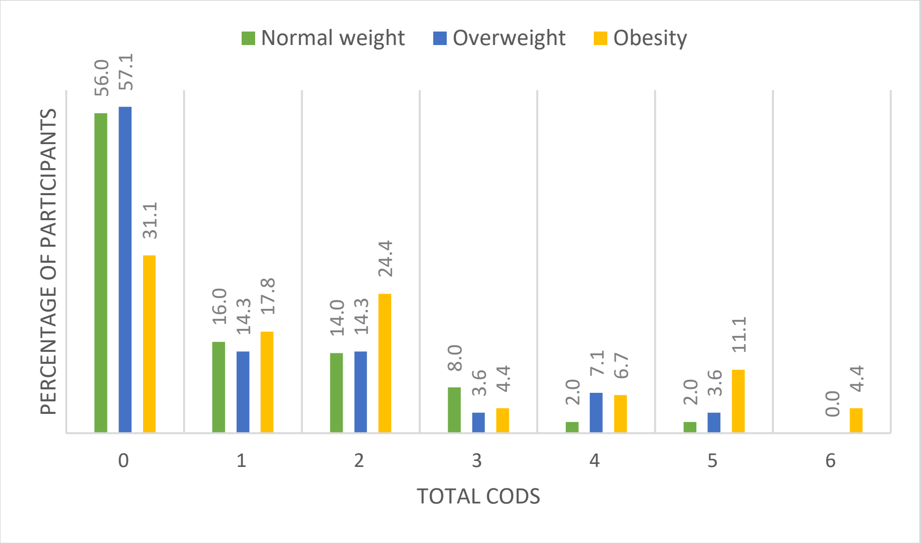

(a)

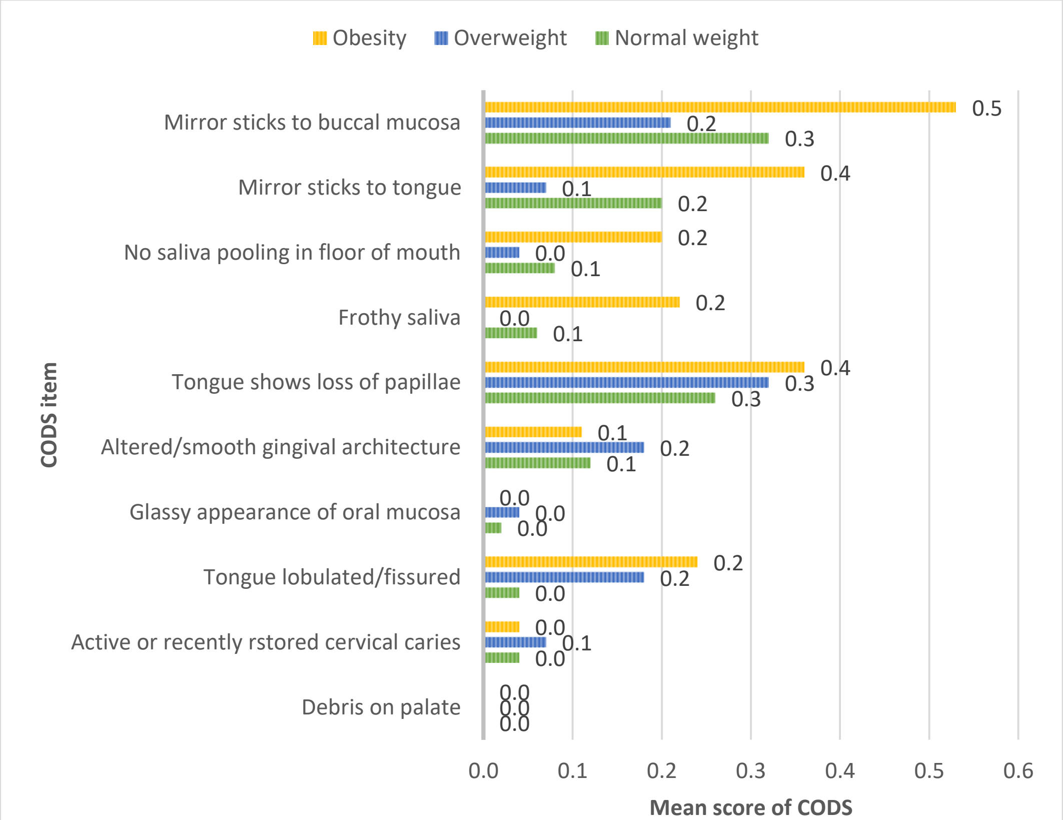

(b)

Figure S2. (a) Percentage of participants according to the total clinical oral dryness score (CODS); **(b)** mean CODS according to each item.
