# Supplementary material for: Increased Oral Dryness and Negative Oral Health-Related Quality of Life in Older People with Overweight or Obesity
Source: Dent J (Basel). 2022 Dec 6;10(12):231. doi: 10.3390/dj10120231 (PMC9776969; doi:10.3390/dj10120231)
Supplement: Supplementary file 1 [file dentistry-10-00231-s001.zip › Figure S4.pdf]

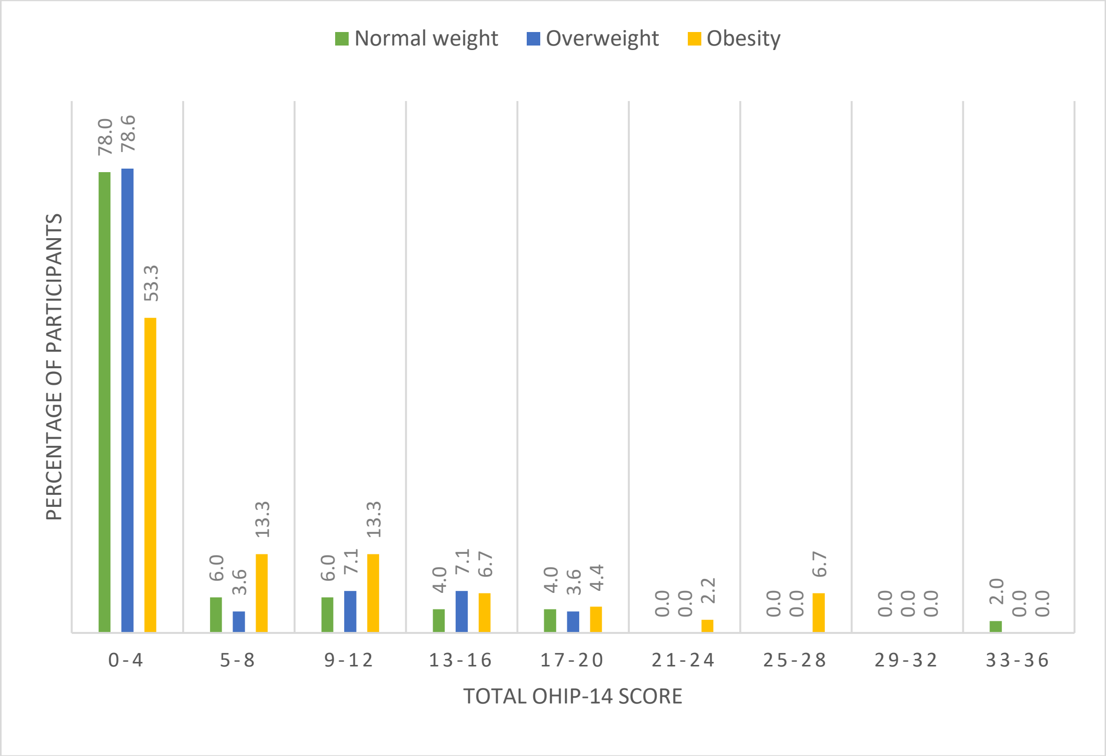

Figure S4. Percentage of participants according to the oral health impact profile (OHIP-14) total score.
