# Supplementary material for: Increased Oral Dryness and Negative Oral Health-Related Quality of Life in Older People with Overweight or Obesity
Source: Dent J (Basel). 2022 Dec 6;10(12):231. doi: 10.3390/dj10120231 (PMC9776969; doi:10.3390/dj10120231)
Supplement: Supplementary file 1 [file dentistry-10-00231-s001.zip › Figure S5.pdf]

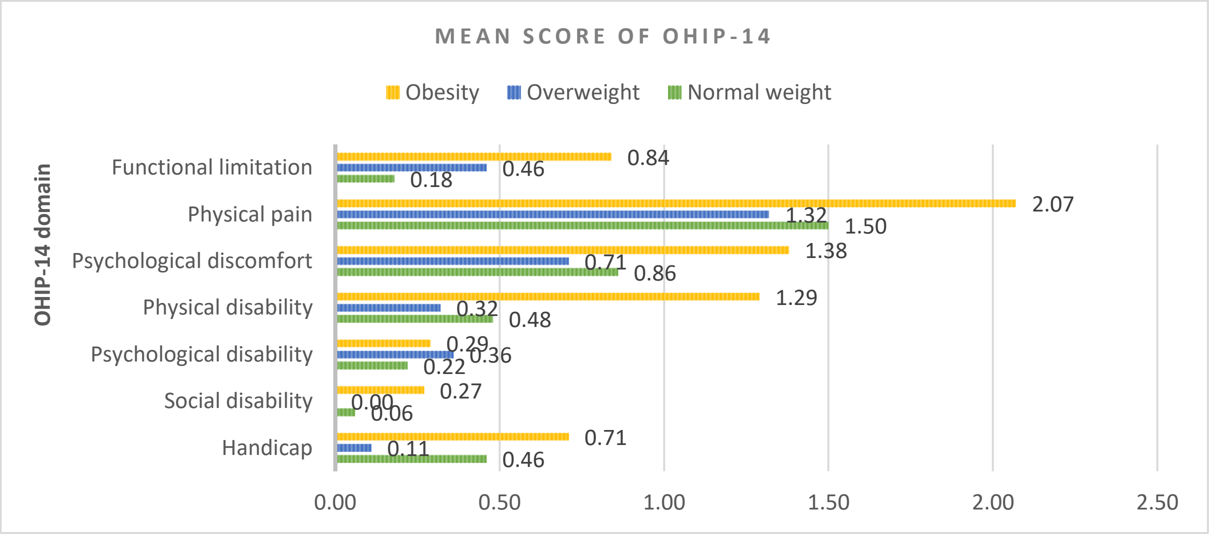

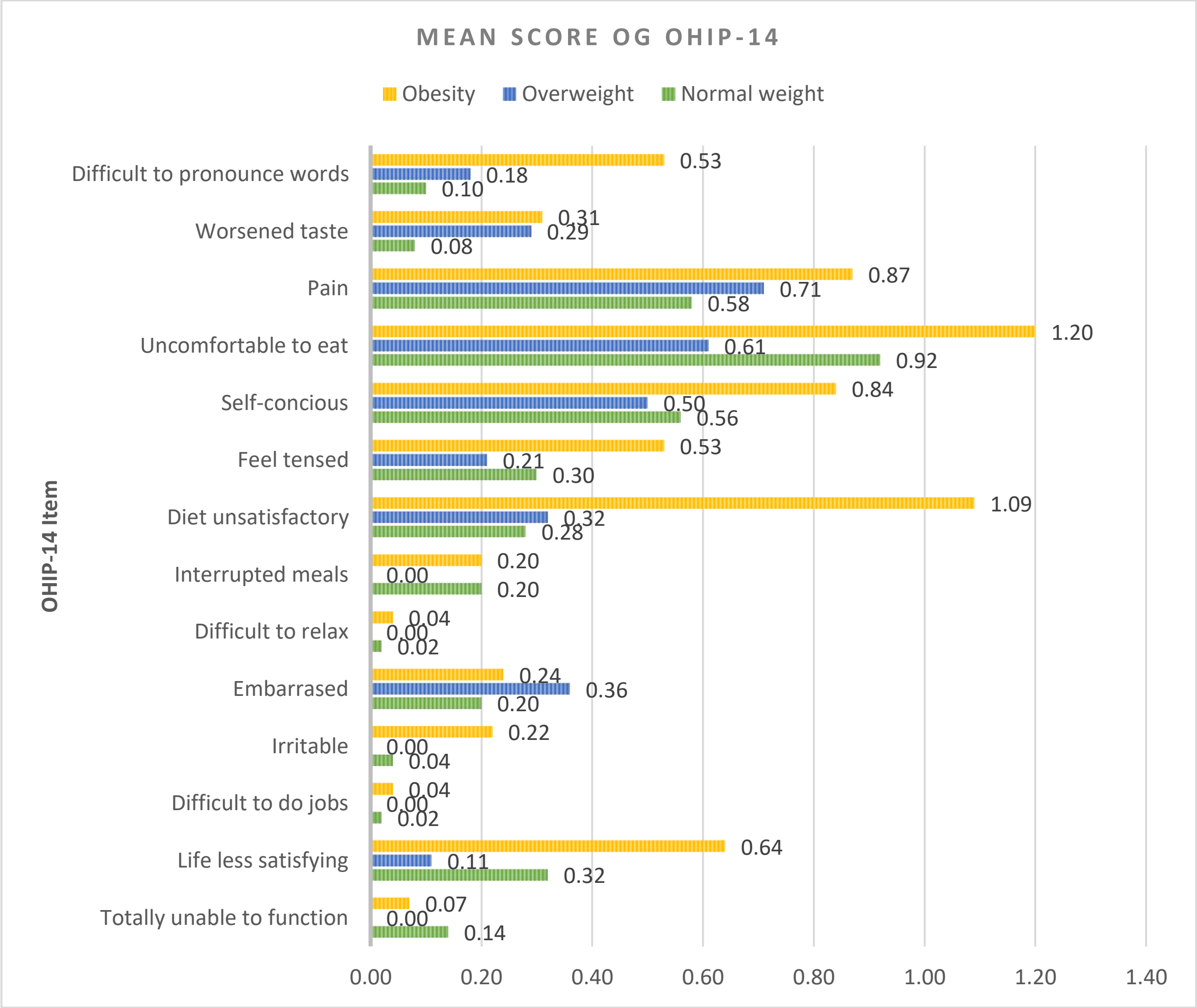

Figure S5. (a) The average severity score of the oral health impact profile (OHIP-14) according to 7 domains; **(b)** The average severity score of the OHIP-14 according to 14 items.
