# Supplementary material for: Increased Oral Dryness and Negative Oral Health-Related Quality of Life in Older People with Overweight or Obesity
Source: Dent J (Basel). 2022 Dec 6;10(12):231. doi: 10.3390/dj10120231 (PMC9776969; doi:10.3390/dj10120231)
Supplement: Supplementary file 1 [file dentistry-10-00231-s001.zip › Table S1.pdf]

Table S1. Family, social, and personal history of participants according to BMI [n (%) or median (first, third quartile)].

	Participants (N=123)			*p*-value
	**Normal weight (n=50)**	**Overweight (n=28)**	**Obesity (n=45)**	
Education				
Less than a bachelor's degree	10 (20.0)	8 (28.6)	15 (33.3)	0.333
Bachelor's degree or higher	40 (80.0)	20 (71.4)	30 (66.7)	
Living				
Alone	5 (10.0)	5 (17.9)	5 (11.1)	0.573
Family	45 (90.0)	23 (82.1)	40 (88.9)	
Health financial status				
Independent	16 (32.0)	8 (28.6)	6 (13.3)	0.233
Dependent	8 (16.0)	7 (25.0)	11 (24.4)	
Welfare	26 (52.0)	13 (46.4)	28 (62.3)	
Dental financial status				
Independent	21 (42.0)	15 (53.6)	18 (40.0)	0.493
Welfare	29 (58.0)	13 (46.4)	27 (60.0)	
Travel				
Independent	50 (100.0)	26 (92.9)	43 (95.6)	-
Dependent	0 (0.0)	2 (7.1)	2 (4.44)	
Exercise				
Less than 3 times/week	23 (46.0)	19 (67.9)	28 (62.2)	0.116
Three times or more/per week	27 (54.0)	9 (32.1)	17 (37.8)	
Alcohol consumption				
Current	16 (32.0)	9 (32.1)	20 (44.4)	0.553
Former	10 (20.0)	4 (14.3)	9 (20.0)	
Never	24 (48.0)	15 (53.6)	16 (35.6)	
Smoking				
Current and former	6 (12.0)	9 (32.1)	14 (31.1)	0.056
Never	44 (88.0)	19 (67.9)	31 (68.9)	
Sleeping hours	6.8 (6.0, 7.5)	6.5 (6.0, 7.0)	6.0 (5.0, 7.0)	0.101
